# Supplementary material for: Variation at Spike position 142 in SARS-CoV-2 Delta genomes is a technical artifact caused by dropout of a sequencing amplicon
Source: Wellcome Open Res. 2021 Nov 10;6:305. [Version 1] doi: 10.12688/wellcomeopenres.17295.1 (PMC9117943; doi:10.12688/wellcomeopenres.17295.1)
Supplement: Peer review history

# Peer review: Reviewer response for version 1

**DOI:** 10.21956/wellcomeopenres.19122.r50103

**Published:** 2022-05-18

**Authors:** Joseph Fauver

**Affiliations:** 1 University of Nebraska Medical Center, Omaha, NE, USA

Sanderson and Barrett take a deep dive into a lineage defining mutation associated with SARS-CoV-2 genomes of the Delta lineage. This was a timely and important piece of detective work that determined there was indeed an amino acid mutation (G142D) in the Spike gene for Delta lineage samples. Their investigation demonstrated that the G->A SNP at nucleotide position 21987 is real and is a clade defining mutation, but that it is often not sequenced due to amplicon dropouts. The ARTIC primer sets have been the workhorse of many SARS-CoV-2 sequencing labs and have been incredibly reliable, however amplicon based approaches are subject to reductions in sensitivities due to mutations in primer binding sites, and in this case a deletion seen in all Delta genomes caused amplicon 72, which covers the site in question, to drop out. The authors also demonstrate that reversions at this site are likely the result of either i) contamination of sequencing libraries with Alpha sequences, or ii) untrimmed primer sequences. Finally, and one of the most useful and interesting points in this manuscript, the authors show that association of genomes with the G142D mutation and a lower CT value/higher viral load is the result of amplicon 72 not dropping out when there is more virus in the sample. There is a correlation between CT value and genome completeness, and in general, more SARS-CoV-2 RNA in a sample will result in more complete genomes, even if there are mutations in primer binding sites. 

 Taken together, this manuscript represents a comprehensive investigation into a pertinent mutation in Delta lineage genomes. My biggest take away from this manuscript is that work like this should be done routinely for all SARS-CoV-2 sequencing projects. Although Delta lineage viruses are no longer circulating, the issues identified here will likely occur again in other lineages at different sites in the genome. All Next Generation Sequencing strategies have associated biases and understanding how those biases and influence consensus sequence genomes and interpretations for clinical or evolutionary outcomes is crucial.

Is the work clearly and accurately presented and does it cite the current literature?

Yes

If applicable, is the statistical analysis and its interpretation appropriate?

Not applicable

Are all the source data underlying the results available to ensure full reproducibility?

Yes

Is the study design appropriate and is the work technically sound?

Yes

Are the conclusions drawn adequately supported by the results?

Yes

Are sufficient details of methods and analysis provided to allow replication by others?

Yes

Reviewer Expertise:

Next Generation Sequencing, genomic epidemiology, virology, parasitology, vector biology

I confirm that I have read this submission and believe that I have an appropriate level of expertise to confirm that it is of an acceptable scientific standard.

# Peer review: Reviewer response for version 1

**DOI:** 10.21956/wellcomeopenres.19122.r47040

**Published:** 2021-11-29

**Authors:** James Davis

**Affiliations:** 1 Consortium for Advanced Science and Engineering, University of Chicago, Chicago, IL, USA

The article by Sanderson and Barrett offers a timely warning about the over the interpretation of SARS-CoV-2 sequencing results. They clearly lay out the cause of the ambiguity for the G142D mutation in Delta variants. I think this needs to be published so that as many people as possible 1) can see the importance of using an up to date primer scheme and 2) can see the importance of submitting sequencing reads to public archives.  

 I found no obvious or major issues with the text.  My only criticism is of the last section of the results, which tries to demonstrate a trend between higher Ct values and ambiguity at position 21987. The two plots look similar to me, especially if they had been plotted as a fraction of the total samples. I think I would need to see statistics to be convinced that these two sets are different (especially since the title of the section is "Increased likelihood... ... ").

Is the work clearly and accurately presented and does it cite the current literature?

Yes

If applicable, is the statistical analysis and its interpretation appropriate?

Partly

Are all the source data underlying the results available to ensure full reproducibility?

Yes

Is the study design appropriate and is the work technically sound?

Yes

Are the conclusions drawn adequately supported by the results?

Yes

Are sufficient details of methods and analysis provided to allow replication by others?

Yes

Reviewer Expertise:

bioinformatics, computational biology, microbiology, sars-cov-2, evolution, machine learning

I confirm that I have read this submission and believe that I have an appropriate level of expertise to confirm that it is of an acceptable scientific standard.
